# Reading the Writing on the Wall: Resident Evaluation Measures as Predictors of Performance in Practice

**DOI:** 10.2106/JBJS.OA.25.00119

**Published:** 2026-09-22

**Authors:** Austin M. Beason, Nicole K. Roberts, Youssef El Bitar, Margaret L. Boehler, Cathy J. Schwind, D. Gordon Allan

**Affiliations:** 1 Division of Orthopaedic Surgery, Southern Illinois University School of Medicine, Springfield, Illinois; 2 Department of Surgery, Southern Illinois University School of Medicine, Springfield, Illinois

## Abstract

**Background:**

Identifying orthopaedic surgery residents at risk of future professional or practice-related difficulties remains challenging. While knowledge-based assessments such as the Orthopaedic In-Training Examination (OITE) are widely used, they incompletely capture behavioral and professionalism concerns that often underlie significant problems during residency and subsequent practice.

**Methods:**

We conducted a retrospective, single-institution cohort study of 119 orthopaedic surgery residents who entered training between 1978 and 2013. Institutional resident files were reviewed for documented performance problems in academic, clinical, and professionalism domains, OITE scores, and American Board of Orthopaedic Surgery (ABOS) Part I and Part II examination outcomes. Adverse postresidency indicators were assessed using publicly available data, including board examination failure, licensure status, practice continuity, and persistent negative online presence. Receiver operating characteristic (ROC) curve analysis was used to evaluate the predictive utility of cumulative documented performance problems and OITE scores.

**Results:**

Forty-two residents (35%) had at least 1 documented performance problem during residency (mean 4.98, range 1-13). Residents with documented problems were significantly more likely to fail ABOS Part I (33% vs. 8%; relative risk [RR] 4.28, p = 0.002) and Part II (24% vs. 4%; RR 6.11, p = 0.004) and to demonstrate persistent negative online presence (19% vs. 5%; RR 3.67, p = 0.026). Having more than 1 adverse postresidency indicator was also more common among residents with documented problems (31% vs. 6%; RR 4.77, p = 0.001). On ROC analysis, 4 or more documented problems predicted failure on ABOS Part I (AUC 0.787) and the presence of adverse postresidency indicators (AUC 0.772), while 3 or more problems best predicted ABOS Part II failure (AUC 0.784). OITE scores demonstrated lower predictive accuracy (AUC 0.696) and lacked a clearly actionable threshold.

**Conclusions:**

Cumulative documented performance problems during residency—particularly behavioral and professionalism-related concerns—are associated with increased risk of board examination failure and adverse practice outcomes after graduation. Aggregated longitudinal documentation may provide an early, complementary signal of risk beyond knowledge-based testing.

## Introduction

Society trusts that residents graduating from accredited orthopaedic surgery programs are competent and capable of delivering safe, ethical, and high-quality care. The American Board of Orthopaedic Surgery (ABOS) advocates for self-regulation and emphasizes our responsibility to our patients is to ensure that board-certified orthopaedic surgeons meet established standards of medical knowledge, patient care, and professional behavior. Currently, without universal criteria defining competence and readiness for independent practice, the sole responsibility for attesting whether a resident is ready to graduate (and thus operate unsupervised) is assumed by the program director relying on the performance data available^1,3^. While tools have been developed to assist program directors assess resident performance in training, they imperfectly identify residents at risk of future difficulties in practice^1,2^.

Objective measures of orthopaedic knowledge, such as the Orthopaedic In-Training Examination (OITE), are well established and have demonstrated moderate correlation with ABOS Part I examination performance. However, knowledge-based examinations alone incompletely capture the behavioral, professional, and clinical integration challenges that account for the majority of problems in residency and in future practice^3-8^.

Residency programs generate extensive longitudinal documentation regarding resident performance, including narrative evaluations, professionalism concerns, remediation efforts, and academic difficulties. Although these data are routinely used for formative feedback and advancement decisions, they are rarely examined in aggregate as potential early signals of long-term risk. Importantly, such documentation has existed for decades, albeit with substantial evolution and structure, terminology, and formality over time.

The primary objective of this study was to examine whether the cumulative number of documented resident performance problems during residency is associated with adverse postresidency indicators, including board examination failure and various markers of practice disruption. A secondary objective was to compare the predictive utility of cumulative problem counts with OITE performance, recognizing the limitations imposed by changes in assessment tools and documentation practices over time. This study was designed as a single-institution, hypothesis-generating analysis focused on identifying behavioral and professionalism signals to predict performance in independent practice.

## Materials and Methods

All residents who matched at a single ACGME-accredited orthopaedic surgery residency program from 1978 to 2022 were included in the study. To ensure adequate postgraduation follow-up, only residents who entered training in 2013 or earlier were included in the final analysis (n = 119). This study was determined to be non‑human subjects research by the IRB (22-256, reference number 029963).

Three experienced medical educators independently abstracted data from institutional resident files into the Research Electronic Data Capture (REDCap; version 14.5.14) database. Formal resident files were maintained throughout the study period; however, the structure, granularity, and terminology of documentation evolved substantially over time. Earlier records relied predominantly on narrative evaluations and program director notes, whereas later records incorporated more structured evaluations. These differences were acknowledged a priori and informed the analytic approach.

### Residency Evaluation Measures

Data collected included information from their ERAS residency application (i.e., unusual comments in letters of recommendation or previous careers), status in the program (graduated or attrition before end of training), OITE score for each training year, number of cases logged, number of attempts on ABOS Part I Qualifying Examination and Part II Certifying Examination, ABOS Part I Examination scores, and any documented performance problems identified by the program director or Clinical Competency Committee (CCC).

Residents who had the same concern documented in their record more than once, or those with multiple documented deficiencies, were considered to have a performance problem. The problems noted in the resident records were categorized using an existing inventory used to assess records for residents in general surgery (Table I)^10^.

**TABLE I:** Residency Problems

Problem	Total No. of Residents
Academic problems	
Insufficient knowledge	20
Putting everything together	5
Clinical problems	
Treatment/Management	14
Case presentation	1
Data interpretation/Diagnosis	6
Technical skill	17
Data collection	2
Intraoperative decision making	7
Lack of preparation	11
Performance under pressure	2
Professionalism problems	
Administrative duties	14
Relations with healthcare workers	19
Time management	9
Communication	6
Lack of self-confidence	4
Inappropriate behavior	9
Teamwork	5
Overconfidence	5
Work avoidance	9
Dependability	10
Relations with patients	8
Dishonesty	6
Lack of apparent interest/Motivation	7
Response to criticism	9
Lack of participation/Withdrawn	2
Documented substance abuse	0

### Performance in Practice

Postresidency records readily available online were reviewed. Google search engine and an internal residency database were used to determine the current practice location for each graduate; graduate licensing status was ascertained using the online state medical license database for the appropriate state. Those records also included some reports of problems such as malpractice claims. Online news reports and patient review sites were also reviewed; patient reviews were only considered to be a problem if there were a significant number of complaints with low ratings (i.e., at least 100 ratings with an average less than or equal to 2.5/5) or if there were egregious complaints.

Adverse outcomes indicative of poor performance in practice included failure to complete the residency program, failure to maintain a medical license, practicing medicine in a nonsurgical specialty, failure to maintain a continuous orthopaedic surgery practice for at least 15 years or without disruption since graduation, and any detrimental mention on licensure sites or online.

### Data Analysis

Descriptive statistics were calculated using SPSS (version 29; IBM). The relative risk (RR) of postresidency adverse outcomes in relation to having significant problems in training was calculated using MedCalc software (version 23.0.6). The OITE score and number of problems identified in residency were subject to receiver operating characteristic (ROC) curve analysis to determine their predictive value of poor performance in practice; a separate ROC curve analysis was performed to assess the role of number of problems in predicting ABOS Part I and Part II performance.

## Results

One hundred thirteen residents graduated from the program and 6 left the program before the end of training (5 residents were dismissed from the program; 1 voluntarily withdrew to pursue a different specialty). Seventy-seven graduates had no documented performance problems in their files, while forty-two graduates (35%) had a performance problem during residency.

Among those with performance issues, the number of distinct problems ranged from 1 to 13 per individual, with a mean of 4.98. The majority of concerns were identified early in training, with 23 residents first flagged in PGY-1 and 16 in PGY-2, while only 3 residents had initial issues noted during PGY-3 or PGY-4. The most common resident problems fell within the domains of professionalism and clinical performance (Table I). Academic problems were also notable, with insufficient knowledge cited in nearly half of cases (n = 20).

Compared with their peers, residents with performance problems were significantly more likely to experience adverse outcomes in independent orthopaedic practice (Table II). Specifically, the residents with documented problems were 4 times more likely to fail the ABOS Part I examination on the first attempt (33% vs. 8%; RR 4.28, *p* = 0.002), over 6 times more likely to fail ABOS Part II (24% vs. 4%; RR 6.11, *p* = 0.004), and more likely to have negative online mentions (19% vs. 5%; RR 3.67, *p* = 0.026). In addition, 18 graduates were noted to have more than 1 negative postresidency performance indicator and having documented problems in residency was a significant predictor (31% vs 6%; RR 4.77, *p* = 0.001). Although increased relative risks were also observed for failure to maintain a medical license, not practicing surgery, and shorter practice duration, these did not reach statistical significance.

**TABLE II:** Negative Postresidency Performance Indicators Among Residents With and Without Performance Problems in Residency

	No Significant Performance Concerns in Residency (n = 77; %)	Performance Problem in Residency (n = 42; %)^†^	Relative Risk (RR)
First-time failure on ABOS Part I (20)	6 (8)	14 (33)	4.28 (*p* = 0.002) *
First-time failure on ABOS Part II (13)	3 (4)	10 (24)	6.11 (*p* = 0.004) *
Not board certified in a surgical specialty (5)	1 (1)	4 (10)	7.33 (*p* = 0.07)
Not practicing in a surgical specialty (5)	1 (1)	4 (10)	7.33 (*p* = 0.07)
Practiced orthopaedic surgery <15 yrs (5)	2 (3)	3 (7)	2.75 (*p* = 0.26)
Online negative mentions (12)	4 (5)	8 (19)	3.67 (*p* = 0.026) *
>1 negative postresidency indicators (18)	5 (6)	13 (31)	4.77 (*p* = 0.001) *

† Residents who had the same concern documented in their record more than once or those with multiple documented deficiencies.

*Statistical significance (p < 0.05).

### ROC Curve Analysis: Resident Problems to Predict ABOS Part I/II Performance

**Fig. 1:**
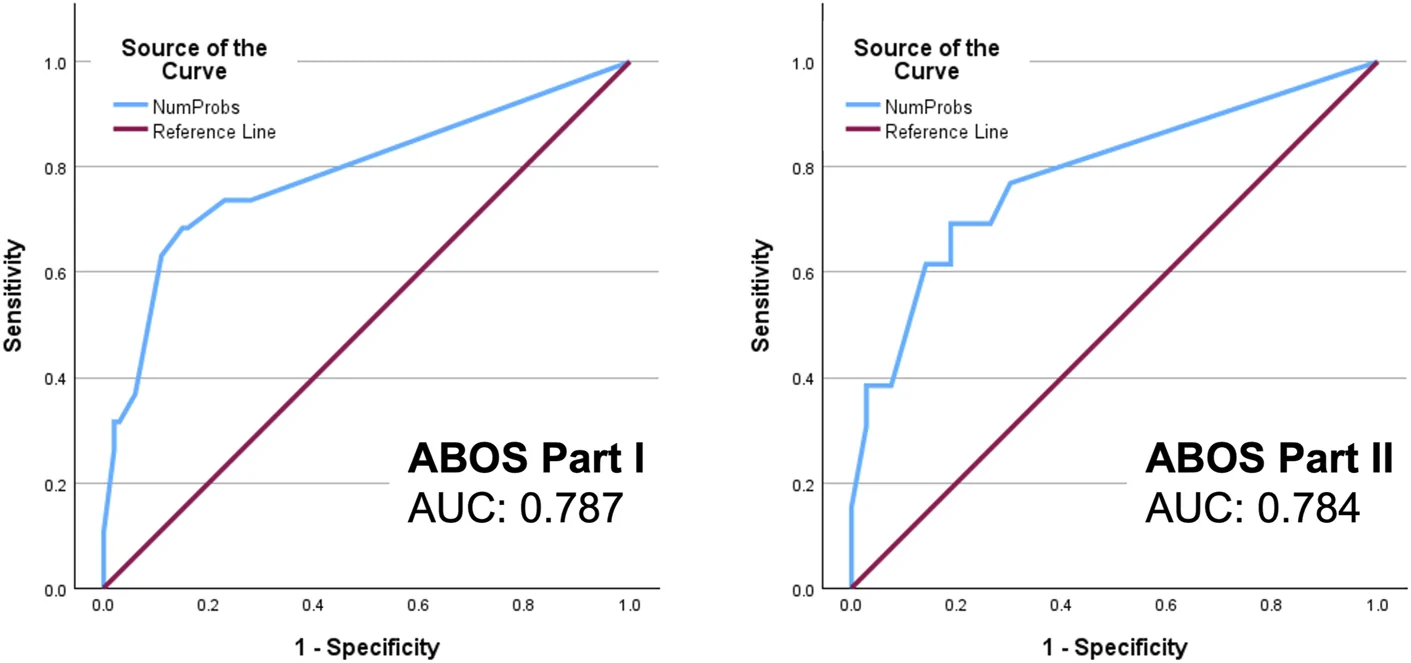
Receiver operating characteristic curves for number of residency problems to predict first-time failure on ABOS Part I Qualifying Examination (left) and Part II Certifying Examination (right). AUC = area under the curve.

Receiver operating characteristic (ROC) curve analysis was performed to evaluate whether the number of performance problems during residency served as a “dose-related” predictor of negative postresidency outcomes, including ABOS board examination failure and suboptimal clinical performance in practice. The area under the ROC curve (AUC) reflects the overall accuracy of a predictive model, where 1.0 represents a perfect test and 0.5 reflects performance no better than chance. The number of problems during training was a strong predictor of board examination failure. Residents with 4 or more problems were at significantly higher risk of failing the ABOS Part I Qualifying Examination, with this threshold providing 68.4% sensitivity and 85.0% specificity (AUC 0.787; 95% CI: 0.656-0.918; *p* < 0.001) (Fig. 1). Similarly, a threshold of 3 or more problems best predicted failure on the ABOS Part II Certifying Examination, with 69.2% sensitivity and 81.1% specificity (AUC 0.784; 95% CI: 0.633-0.935; p < 0.001) (Fig. 1). In practical terms, residents accumulating 3 or more documented performance problems during training should be considered at elevated risk of board examination failure.

#### ROC Curve Analysis: Resident Problems and OITE to Predict Performance in Practice

**Fig. 2:**
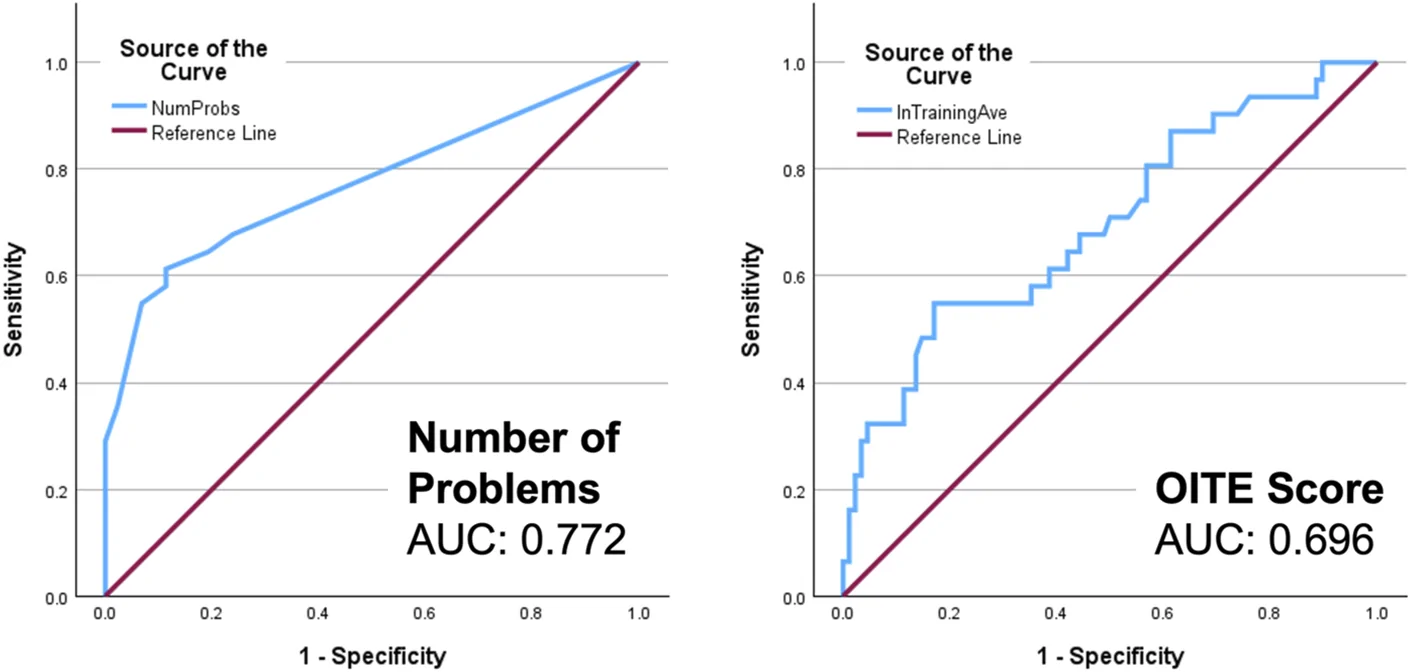
Receiver operating characteristic curve for total number of problems (left) and OITE score (right) to predict poor postresidency performance. AUC = area under the curve, and OITE = Orthopaedic In-Training Examination.

When compared with OITE scores, the number of residency problems was found to be a stronger predictor of postresidency performance, with 4 residency problems being the critical threshold for predicting having an adverse outcome indicating poor performance in practice (AUC 0.772; sensitivity, 58%; specificity, 89%) (Fig. 2). OITE scores were only moderate predictors (AUC 0.696) and lacked a clearly actionable threshold (Fig. 2).

## Discussion

The “Holy Grail” of competency assessment is to predict which residents will provide patient care that is safe and ethical, and simultaneously, to identify residents who will struggle in practice so they can be helped to remediate deficiencies while in the training environment.

The relationship between the Orthopaedic In-training Examination (OITE) and ABOS Part I has been explored extensively, and the effort to improve correlation between the two is ongoing^11-16^. Fritz et al. demonstrated that residents with a surrogate “passing” OITE score had ABOS Part I pass rates of 99.8% in 2022 and 99.7% in 2023, highlighting the strong positive predictive value of the linking efforts; however, 89.9% of residents who fell below the threshold (i.e., had surrogate OITE “failing” scores) still passed the ABOS Part I examination, underscoring the role of the OITE as a valuable but not definitive tool for identifying at-risk examinees^13^. Our findings suggest that other resident assessment measures outperform the OITE in terms of predicting performance in practice. Resident problem counts provide clear, actionable thresholds, which may serve as a complementary objective tool to be used with OITE scores by program directors to move beyond subjective concern toward targeted academic support and data-informed decision making^14^.

The majority of performance problems our residents were cited for during residency were related to professionalism, and the most common reason for resident dismissal from a program according to a study by Clark et al. is poor professional behavior^17^. Unprofessional behavior can lead to increased risk of surgical and medical complications^18^, and as we engage in self-regulation as a profession, the risk of patient harm demands our attention. Our findings are consistent with other literature regarding professional behavior that has demonstrated medical students who are called before their disciplinary committee for professionalism concerns are at risk of being disciplined in residency and in practice^5,6,19^. “Relations with healthcare workers” was the second most prevalent resident problem in our cohort, and as our profession continues to grapple with how to manage the disruptive physician, it is evident that early detection and remediation is beneficial^3,20^. The development and validation of the ABOS Behavior Tool (ABOSBT) allows for identification of “outlier” residents and will help bring objectivity to an area of assessment that is traditionally highly subjective^9^. However, in its pilot study, most residents demonstrated excellent professional behavior across all domains on the ABOSBT regardless of the training level, which is in contrast to knowledge and clinical skills that tend to improve over time^9^. The relatively static nature of these ratings highlights the need for early identification of issues in professionalism^5,6,9^.

We acknowledge several limitations of our study. Our data were collected retrospectively and are therefore limited to existing records. While we recognize the dramatic change in assessment tools and methods used to assess resident performance throughout the study period, the problem categories have remained consistent over time (e.g., insufficient knowledge, relations with healthcare workers, and technical skill deficiency). The problems cited in the residents’ files were categorized by the authors and reflect our interpretation; however, our findings suggest that 4 problems, regardless of category, predict negative postresidency performance. The authors acknowledge that the problem list we used to organize and tabulate problems is not validated; however, the domains align with nationally accepted competency standards in orthopaedic surgery education (i.e., the ABOS Knowledge, Skills, and Behavior Program), and the specific problems are common across programs. Defining performance in practice is inherently subjective and cannot be distilled down to a short list of indicators. Nonetheless, losing a medical license, failing board certification, being unable to maintain a surgical practice, receiving poor patient reviews, or incurring malpractice claims are generally accepted signs of poor performance. Although patient outcomes would contribute meaningfully to defining performance, these data were unavailable and may add further subjectivity if relying on patient-reported outcomes. Future research should explore external validation across multiple residency programs and investigate whether structured performance tracking combined with emerging tools such as natural language processing of narrative evaluations or AI-based risk stratification can improve predictive accuracy and support early intervention.

In 1984, the AOA sponsored an ad hoc Committee on Resident Selection and found that 1 in 6 resident selections made by program directors was deemed inappropriate and that 1 in 12 was deemed to be a serious mistake^3,21^. While this may seem historical in nature, nearly 20 years later, Michael Simon noted in his 2001 Presidential Address to the AOA that ABOS oral examination candidates reported that 6.3% to 12.2% of practicing orthopaedic surgeons in their communities should not be certified due to unethical conduct. In response, the AAOS sponsored an ad hoc Committee on Troubled Physicians that reached the conclusion that surgeons in practice who failed ABOS Part II could have readily been identified as problem residents potentiating early remediation or termination in training^3^. Residency programs should heed this cautionary tale and closely monitor their residents for predictors of future problems if they are to avoid introducing graduates into independent practice and allowing history to repeat itself.

## Conclusions

The cumulative number of resident problems is a strong, objective predictor of board failure and suboptimal practice performance. Receiver operating curve analysis identified residents with 4 or more different problems in residency were more likely to have negative postresidency indicators (i.e., poor performance in practice), and those with 3 or more problems were at risk of failing ABOS Part I and Part II. While the sensitivity and specificity of these problem counts are insufficient to make definitive decisions, they are sufficiently indicative to allow the program director to discuss serious remediation needs and the “goodness of fit” of the orthopaedic profession for the resident.
